# Getting your game on: Using virtual reality to improve real table tennis skills

**DOI:** 10.1371/journal.pone.0222351

**Published:** 2019-09-10

**Authors:** Stefan Carlo Michalski, Ancret Szpak, Dimitrios Saredakis, Tyler James Ross, Mark Billinghurst, Tobias Loetscher

**Affiliations:** 1 Cognitive Ageing and Impairment Neurosciences Laboratory, School of Psychology, University of South Australia, Adelaide, South Australia, Australia; 2 Empathic Computing Lab, School of Information Technology and Mathematical Sciences, University of South Australia, Adelaide, South Australia, Australia; The Education University of Hong Kong, HONG KONG

## Abstract

**Objective:**

The present study investigates skill transfer from Virtual Reality (VR) sports training to the real world, using the fast-paced sport of table tennis.

**Background:**

A key assumption of VR training is that the learned skills and experiences transfer to the real world. Yet, in certain application areas, such as VR sports training, the research testing this assumption is sparse.

**Design:**

Real-world table tennis performance was assessed using a mixed-model analysis of variance. The analysis comprised a between-subjects (VR training group vs control group) and a within-subjects (pre- and post-training) factor.

**Method:**

Fifty-seven participants (23 females) were either assigned to a VR training group (*n* = 29) or no-training control group (*n* = 28). During VR training, participants were immersed in competitive table tennis matches against an artificial intelligence opponent. An expert table tennis coach evaluated participants on real-world table tennis playing before and after the training phase. Blinded regarding participant's group assignment, the expert assessed participants’ backhand, forehand and serving on quantitative aspects (e.g. count of rallies without errors) and quality of skill aspects (e.g. technique and consistency).

**Results:**

VR training significantly improved participants’ real-world table tennis performance compared to a no-training control group in both quantitative (*p* < .001, Cohen’s *d* = 1.08) and quality of skill assessments (*p* < .001, Cohen’s *d* = 1.10).

**Conclusions:**

This study adds to a sparse yet expanding literature, demonstrating real-world skill transfer from Virtual Reality in an athletic task.

## Introduction

Using Virtual Reality (VR) as a tool for training is becoming increasingly popular. VR is an immersive digital space where users can interact with objects and navigate as if they are present in a real environment [1, 2]. VR technology has garnered interest for use in situations where training in the real world is logistically difficult to organise, dangerous or impractical [2]. For example, training programs for surgeons [3], pilots [4] and firefighters [5] are taking advantage of the realism and flexibility that VR offers. A key assumption of VR training is that the learned skills and experiences transfer to the real world [6]. Yet, in certain application areas, such as VR sports training, there has been little research testing this assumption.

Applying VR to sports training has several advantages. Firstly, it offers the possibility for people to train without needing access to the necessary sporting environment (e.g., a downhill slope for skiing) or multiple training partners (e.g. football). Secondly, actively incorporating VR into sports training allows users to log their performance and closely monitor their development. Thirdly, VR is scalable and provides a great degree of freedom to create and control virtual environments in diverse ways. These advantages provide an immense opportunity to incorporate evidence-based practices to achieve greater performance gains from training. For example, evidence-based research on training, such as the challenge point framework, suggests that individuals develop their skills best when they are constantly challenged [7]. Integrating this research into a virtual environment can easily be accomplished by adding variability into training and systematically adjusting the level of difficulty based on the ability of the user [8].

There are many examples of training in VR which has led to positive real-world outcomes. For example, when medical students used a medical VR training simulator to learn gallbladder dissection, they were able to perform the task 29% faster in the real world than non-VR trained students and with six times fewer errors [3]. Bliss et al. [9] found that VR could be used to train firefighters equally as well on real world spatial navigation as traditional methods, and Carlson et al. [10] found that VR training improved performance on a real world assembly task. However, researchers such as Kozak et al [11] found that there was no transfer of training from VR to the real world, in this case using a pick and place task. These conflicting results show that there is still more research that needs to be done on transfer of training from VR to the real world.

Similarly, for VR to be considered as an effective tool for sports training, positive transfer from virtual training to real-world performance must be demonstrated [12]. Todorov, Shadmehr and Bizzi [13] were one of the first to investigate transfer of sports training in a virtual environment. In their first experiment, a virtual training group practised table tennis on a computer monitor placed next to a real table tennis table. In real-time, the monitor relayed a virtual table, ball, and an expert’s paddle which was used to demonstrate ideal movement strokes throughout the training [13]. During virtual training, participants were required to imitate the ideal table tennis strokes from the expert’s paddle while they attempted to hit targets on the real table tennis setup.

Todorov and colleagues [13] found that target accuracy significantly improved in the virtual training group compared to a group that received coaching from a table tennis professional. Despite the virtual training group’s improvements in target accuracy, their overall technique deteriorated compared to the ideal movement strokes for this task. Notably, the real-world coaching group did not experience the same negative effects on their technique, even though their accuracy was substantially lower than the virtual training group. Todorov et al.’s [13] study poses an intriguing dilemma for VR training as their virtual training group exhibited both positive and negative transfer.

Although positive transfer is the best outcome for virtual training, sometimes negative transfer can happen when the virtual environment does not accurately represent the task used for training [6]. Inconsistencies in a virtual training environment can lead to users experiencing unnatural movements, which may hinder their performance in the real-world version of that task. In the case of Todorov et al.’s [13] study, the training task was uncharacteristic as participants had to focus on a computer screen while trying to connect with a ball and hit a target on a real table. Negative transfer may have occurred in this study, as participant’s attention was divided as it was directed towards the screen—vastly different from what occurs during table tennis in the real world.

Assessing transfer in a sport can be challenging when there are many qualities of performance improvement to consider. For example, Todorov et al.’s [13] study had several limitations regarding transfer assessments as they overlooked key table tennis skills, namely; serving, backhand and forehand. Attaining a reliable index of a player’s performance and transfer requires consideration of both quantitative aspects (e.g. serving accuracy, performance in rallies) and overall quality (e.g. technique, consistency, coordination).

By nature, table tennis is a sport which requires open skills. Open skill sports are those in which players must respond in a continually changing, unpredictable and externally-paced environment [14], typically involving the presence of an opponent (e.g., as in table tennis, football, boxing, etc.). Specifically, table tennis requires flexibility in visual attention, quick decision-making and fast interceptive actions in response to an interactive opponent [15–17]. In contrast, closed skill sports involve a predictable, relatively consistent and self-paced environment (e.g., golf, cycling, darts, etc.) [14]. The results from Todorov and colleagues [13] provide support that training in a virtual environment can improve closed table tennis skills (target accuracy). While there is some research to support the notion that basic closed skills are transferable from VR to the real-world [3, 8, 13], it is unknown whether VR can be used to develop the more complex open skills, which are essential in sports such as table tennis.

It is important to consider how the training conditions can be structured to maximise the opportunity for improvement [7]. More recently, Gray [8] conducted a study investigating transfer in VR baseball training. A group that received repetitive batting practice in the real world was compared to a group that received repetitive batting practice in a virtual environment, and a group that received adaptive training in a virtual environment. Gray [8] found that when training was adaptive (adjusted to the level of success during training), real-world performance significantly improved in comparison to both groups that received only repetitive practice [8]. Adaptive training is based on the premise that, for learning to occur, there is an ideal amount of information necessary which varies as a function of the individual’s level of skill and difficulty of the task in training [7]. Perhaps, the value of VR training is not to recreate real-world training, but to implement principles of evidence-based practice (e.g. adaptive training) that is difficult to structure into real-world training.

Despite these positive results, for VR to become an accepted tool in sports training, it must be determined whether it can have a real-world impact. The present study aimed to investigate the transfer effects from VR training to the real world, using the fast-paced sport of table tennis. It was hypothesised that VR table tennis training will lead to significant improvements (pre-test to post-test) in real-world performance (serving and rallying) on both quantitative aspects (serving accuracy and number of rallies without errors) and overall aspects of skill quality (ball height, strength, consistency, technique and coordination) as compared to no training.

## Method

### Participants

Sixty-two English-speaking people between 18 and 35 years of age participated in this study. Five participants were excluded from the final analysis due to; withdrawal (*n* = 3), playing table tennis outside of the study (*n* = 1) and scoring above 90% on the quantitative assessment at pre-test (*n* = 1), with insufficient room for improvement.

Thus, a total of fifty-seven participants were included in the analyses (*M*_*age*_ = 21.81, *SD* = 3.58). Participants were divided into either a VR training group (*n* = 29) or a control group (*n* = 28). The key characteristics of participants in the VR training group and the control group are illustrated in Table 1.

**Table 1:** Key characteristics of participants in the VR training group and control group.

Variable	VR training	Control
*n*	29	28
Mean age (± *SD*)	22.07 (4.27)	21.54 (2.75)
Gender	Males = 18 Females = 11	Males = 16 Females = 12
Hand preference [18]*	Right (*n* = 29)	Right (*n* = 28)
Mean days from pre-test to post-test (± *SD*) **	25.17 (6.86)	23.25 (3.58)

*Hand preference was determined using the Flinders Handedness Survey.

**Independent samples t-tests revealed no significant group differences the number of days from starting to completing the study (pre-test to post-test), *t*(55) = 1.31, *p* = .190.

No participant had an injury, disability or any other reason which could affect their involvement in the study. To ensure that participants had normal or corrected-to-normal visual acuity their vision was assessed on various tests including: *Snellen Eye Chart*, *RAF Rule* and *Fonda-Anderson Reading Chart*. In addition, participants in the VR training group had normal or corrected-to-normal stereo acuity, as determined by the *Butterfly Stereo Acuity test* (Vision Assessment Corporation, 2007). None of the participants were competitive table tennis players.

Participants were recruited via flyers which were placed around the University of South Australia (UniSA) Magill Campus. All participants provided informed consent prior to participation and received an honorarium of $20/hour for participation. The study was granted ethics approval from the UniSA Human Research Ethics Committee.

The appropriate sample size was calculated using the G*Power3 software [19]. Experiment 1 in the study by Todorov et al. [13], examined transfer of table tennis skills in a virtual environment via number of target hits before and after virtual training, finding an effect size of 0.86. Using this effect size as an estimate for the Power Analysis, it was calculated that 18 participants per condition would be needed to suffice power (0.80) with *α* = 0.05.

### Materials and apparatus

#### VR apparatus

The HTC Vive head-mounted display (HTC, with technology by Valve Corporation, April 2016) was used. Immersive stereoscopic head-mounted displays (HMD) such as the Vive create a sense of presence in the user by viewing a 360-degree virtual environment that moves in real time in accordance with the movements of the participant [20]. Users were required to wear the HMD while holding two controllers to interact within the virtual environment, one as a device to simulate ball dispersion and another as a simulated table tennis bat. The HMD accommodates users with visual impairments, by allowing them to wear glasses and contact lenses while using the device. The VR apparatus was used in a 1930mm x 3300mm room with two base stations installed in opposite corners, enabling room-scale tracking.

#### VR table tennis game

Eleven: Table Tennis VR (developed by Fun Labs) was used. This is a game which requires users to interact by moving and responding to incoming stimuli. Users are immersed in a competitive game of table tennis against an AI opponent, to which official table tennis rules apply. The difficulty of the AI ranged across five levels from amateur to legendary, ascending in order of difficulty. An increase in difficulty is related to an increase in the speed of serve and return, number of serve/return placements and spin on the ball from the AI. The game utilised haptic, auditory and performance feedback to simulate a real-world environment. For example, when the simulated bat hit the ball users felt a vibration and, realistic sounds were played. Sounds were also used to signal a won or lost point while a scoreboard was also available.

#### Real-world table tennis setup

The table tennis setup included a regulation size STIGA table tennis table, Dawei table tennis bats and 40mm Schildkrot table tennis balls. Ten empty standard size soft drink cans were used as serving targets.

### Assessment and measures of real-world table tennis performance

#### Assessor

An expert table tennis player credentialed with international medals and over 40 years of experience as a table tennis coach was employed. The assessor did not know the participant’s group assignment throughout the study. The assessor had a critical role in developing the real-world table tennis tasks, which were based on training drills used by table tennis coaches. The quantitative assessment and the quality of skills assessment were developed based on an established grading system the assessor uses in coaching.

#### Quantitative assessment

A quantitative score was derived for each participant based on their performance in the table tennis tasks. Scores were calculated based on the number of successful returns made in each of the three rallying tasks: backhand, forehand and alternating hits (consecutively changing from one hit of forehand to one hit of backhand). A successful return is when the ball lands on your side of the table, then it hits the bat, then lands on the opponent’s side of the table. Each participant had three attempts per rallying task, and a score was averaged based on the highest two attempts, to improve reliability of the assessments. For backhand, forehand and alternating hits, scores ranged from 0 to 100 with a higher score indicating a higher number of successful returns made. In these assessments, one score is equal to one successful return. If a participant were to reach 100 returns, the rally ended.

In addition to the rallying tasks, serving accuracy was assessed via target accuracy. The targets were ten soft drink cans which were evenly distributed (100mm apart) on the edge of the opposite end of the table. This was used as the target as it is recommended that an ideal serve is one that reaches close to the edge of the opposite end of the table, while bouncing as low as possible off the table. A score was derived based on the number of targets a participant could hit while serving, ranging from 0 (no target hit) to 10 (all targets hit). Each target hit was worth 5 points. A total score was calculated for each participant from 0 to 350 (backhand = 100, forehand = 100, alternating hits = 100, serving = 50). Participants were excluded from the study if they achieved a total score above 90% at pre-test, as it was deemed there was insufficient room for improvement.

#### Quality of skills assessment

The quality of skills assessment was based on the assessor’s observations of each participant in the table tennis tasks. The assessor evaluated the improvement in each participant’s quality of skills based on five categories: ball height, strength, consistency, technique and coordination. During pre-test the assessor formulated extensive notes on the quality of these five different skills, creating a baseline for each participant. The assessor often formulates notes in a similar respect during external coaching. The assessor referred to these baseline notes in the post-test session to determine any improvements in the quality of skills since the pre-test session. The assessor scored improvements in each of the five categories with a score of either 0 (no improvement), 1 (some improvement) or 2 (major improvement). A total score was calculated from 0 to 10 based on the sum of scores of all five categories, with higher scores reflecting a greater improvement in skill quality.

As both the quantitative assessment and the quality of skills assessment were assessing different aspects of table tennis skills, it was expected that an individual might improve on one assessment, while not on the other. For this reason, both measures were considered independently in analysis.

### Design

**Fig 1:**
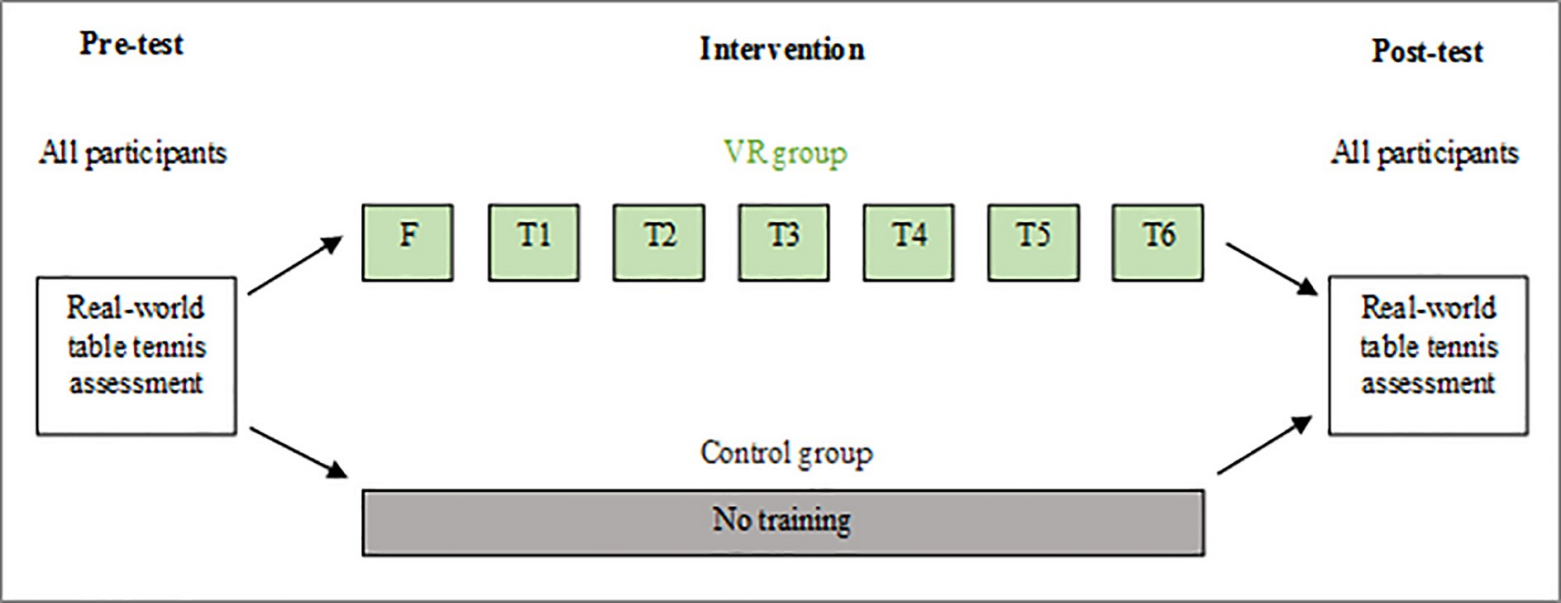
Outline of study design. There were two participant groups: VR group and control group. All participants underwent a pre- and post-assessment. Participants in the VR group completed a familiarisation (F) session followed by six VR training sessions (T1-T6).

Real-world table tennis performance was assessed in the VR training and control groups before and after the intervention phase. See Fig 1 for an outline of the study design.

The study hypothesis was addressed using a mixed-model analysis of variance. The analysis comprised a between-subjects (VR training group vs control group) and a within-subjects (pre- and post-training) factor. The dependent variable was real-world table tennis performance assessed by an expert on both quantitative aspects and skill quality aspects.

### Procedure

Each participant was first guided through the consent process. All participants then completed several questionnaires which included questions regarding the participants’ demographics (e.g., age, gender, and handedness). They then underwent visual assessments to ensure normal or corrected-to-normal visual and stereo acuity.

Participant’s table tennis skills before and after the intervention (VR training or no-training) were assessed on a real table tennis setup, by an expert table tennis coach. Blinded regarding participant's group assignment, the expert assessed quantitative and quality aspects of participants’ table tennis performance in pre- and post-tests. The study design for both participant groups is summarised in Fig 1. Participants were asked to not play table tennis at any time other than what was required for participation until completion of the study. Cognitive and further visual assessments were administered to participants in the familiarisation session; however, these data are not relevant for the purpose of this study and not reported here (see: Szpak et al. [21] for more details on the cognitive and visual assessments).

#### Pre-tests

Participants started with five minutes of rallying practice with the assessor. This was designed for participants to warm up. During this practice period, the assessor provided brief instructions regarding the correct rules of table tennis. Participants did not receive any tips/advice on how to improve.

Immediately after the practice, participants completed the forehand, backhand and alternating rallying tasks described in the Assessment and Measure of Real-World Table Tennis section. Participants had three attempts in each of the three tasks. The participants were told to rally and to return the ball as many times as possible in a row with the assessor. It was made clear that the task was not about winning the point.

During the forehand task, the stroke of the participant must have been played with the palm of the hand facing in the direction of the stroke. During the backhand task, the stroke of the participant must have been played with the back of the hand facing in the direction of the stroke, with the arm across the body. If during either assessment the participant hit the ball in any other capacity the rally was ended. The alternating forehand and backhand task consisted of hitting a ball once with backhand, then once with forehand, continuing this pattern throughout. During this task, if the participant hit the ball, either forehand or backhand twice in a row, the rally ended. During all three tasks, if a participant attempted a return which missed their bat or did not hit the assessor’s side of the table, the rally ended. If the assessor happened to unsuccessfully return the ball to the participant, a new rally was started instantly by the assessor and the rally continued from that point onwards.

Once the rallying tasks were complete, the participants began a serving task which involved hitting targets. The aim expressed to the participants was to either hit or knock down the targets. Participants had ten opportunities to serve to hit the ten targets. If the target was hit with an illegal serve the target was re-positioned and the score was not included, however, it was considered as one of the ten attempts. Prior to this task, participants were offered three minutes of target practice. Participants received no coaching or feedback during or after completion of the tasks.

### Intervention

**VR training group:** All VR training was completed using the HTC Vive HMD and the Eleven: Table Tennis VR application. During training, participants experienced relevant tasks that were assessed during pre-tests and post-tests including serving, backhand and forehand. Each participant in the VR training group completed a total of 3 hours and 30 minutes in VR, split across seven sessions. It was recommended that participants completed two sessions per week. Participants could complete only one training session per day.

First, there was a familiarisation session. During this time, participants received a tutorial with the VR apparatus and table tennis game, as well as instruction regarding the subsequent training. Participants had the remainder of this session to practice and were informed scores were not assessed. The familiarisation was introduced to make sure participants understood the task and tolerated VR exposure.

The six VR training sessions aimed to beat the AI opponent in games of table tennis. The winner of a game was the first get 11 points. For the game to be won the victor had to be up by 2 points, thus games could exceed 11 points. Upon completion of a game, a new one commenced. The training involved best-of-five series. Using an adaptive training procedure, a participant winning the series (i.e. winning three out of five games) would be moved up on difficulty level, a participant losing the series (i.e. lose three out of five games) would be moved down one difficulty level or stay on the lowest level if they were already there. Each training lasted for approximately 30-minutes. Best-of-five series that were incomplete at the end of training were continued in the next training session.

**Control group:** The control group did not receive any training during the intervention phase.

#### Post-tests

All participants completed the post-test which was identical to the table tennis tasks in the pre-test. After the post-test, the researcher asked participants if they played table tennis at any other time since starting the study and were excluded from the study if they did for over one hour in total.

## Results

### Quantitative assessment

**Fig 2:**
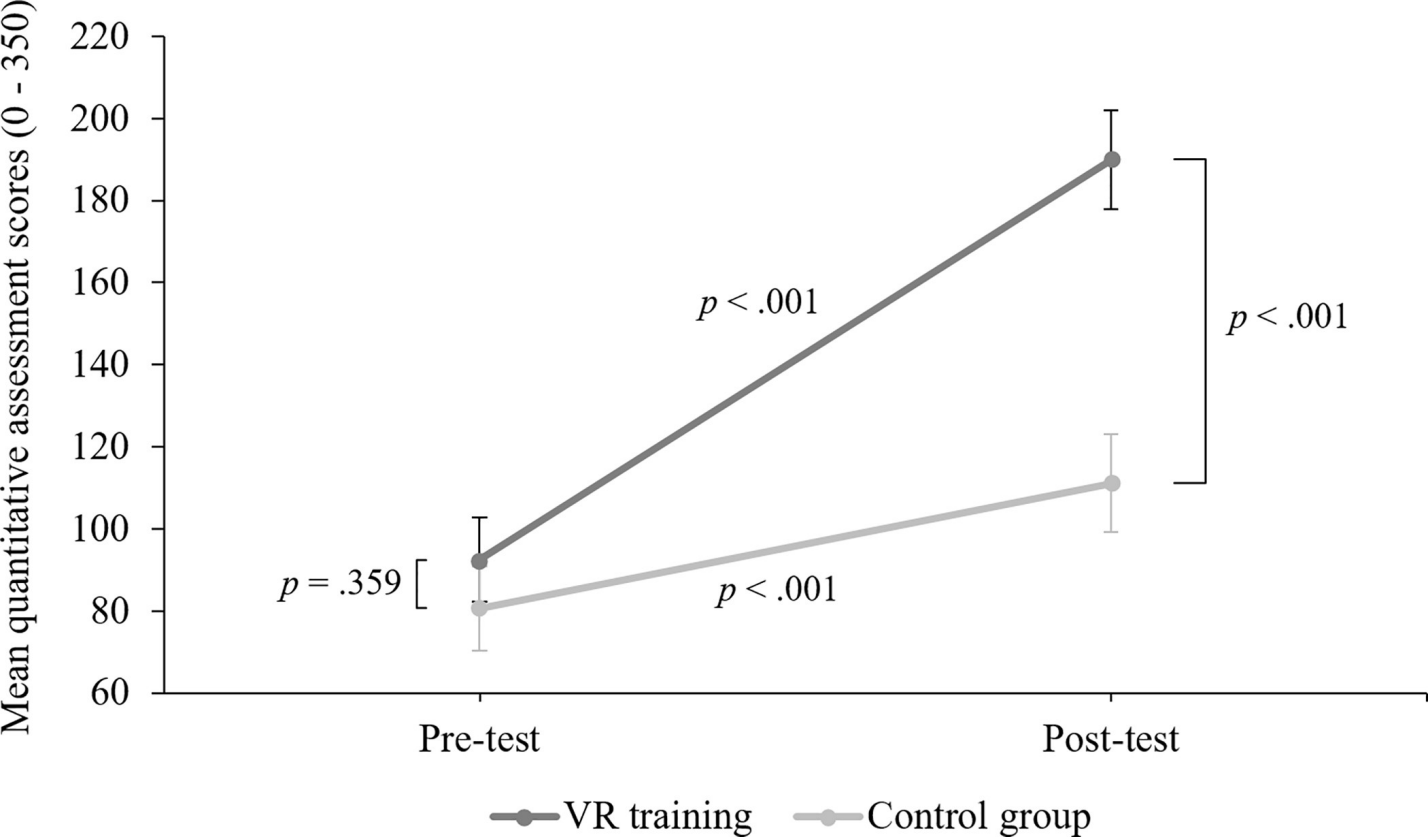
Quantitative assessment. Total Mean (± standard error) scores on the quantitative assessment for the VR training group (dark grey) and the control group (light grey) at pre-test and post-test.

A mixed ANOVA was conducted with time (pre-test and post-test) as a within-subjects factor and group (VR training and control) as a between-subjects factor. This test met the homogeneity of variances assumption as Levene’s Test was not significant. Thus, equal variances were assumed. A significant main effect of time (*F*(1, 55) = 86.47, *p* < .001. Partial eta^2^ = .611) and group (*F*(1, 55) = 9.31, *p* = .003. Partial eta^2^ = .145) were found. There was also a significant interaction effect between time and group *F*(1,55) = 23.66, *p* < .001. Partial eta^2^ = .301, see Fig 2.

To further investigate the significant interaction, a series of Bonferroni corrected post hoc t-tests were conducted. Bonferroni adjustments for multiple comparisons of four variables (*a* = .0125) were applied. Paired samples *t*-tests revealed that both the VR training and the control group significantly improved their real-world table tennis performance from pre to post-test. The scores of the VR training group improved from *M* = 92.46 (*SD* = 42.25) at pre-test to *M* = 189.93 (*SD* = 80.68) at post-test (*t*(28) = -7.8, *p* < .001. Cohen’s *d* = 1.70). The scores of the control group improved from *M* = 80.62 (*SD* = 53.93) at pre-test to *M* = 111.14 (*SD* = 63.47) at post-test (*t*(27) = -5.6, *p* < .001. Cohen’s *d* = 0.51).

An independent samples *t*-test revealed there were no significant differences between the VR training group (*M* = 92.46, *SD* = 42.25) and the control group (*M* = 80.62, *SD* = 53.93) at pre-test on the quantitative assessment, *t*(55) = .92, *p* = .359.

An independent samples *t*-test revealed at post-test, participants in the VR training group (*M* = 189.93, *SD* = 80.68) scored significantly higher than participants in the control group (*M* = 111.14, *SD* = 63.47) on the quantitative assessment, *t*(55) = 4.08, *p* < .001. Cohen’s *d* = 1.08.

#### Test-retest reliability

Test-retest reliability was assessed by correlating the pre- and post-test scores of the control group. Based on Hopkins [22] the intraclass correlation coefficient (ICC 3,1) was 0.89. The ICC thus indicates good test-retest reliability.

### Quality of skills assessment

An independent samples t-test revealed that the change in scores for participants in the VR training group (*M* = 7.37, *SD* = 2.24) were significantly higher than participants in the control group (*M* = 4.46, *SD* = 2.97) on the quality of skills assessment, *t*(55) = 4.18, *p* < .001. Cohen’s *d* = 1.10.

The scores of the VR training group (*M* = 7.37, *SD* = 2.24, (*t*(28) = 17.71, *p* < .001)) and control group (*M* = 4.46, *SD* = 2.97, (*t*(27) = 7.94, *p* < .001)) were significantly different from zero (i.e. a score of zero means no change from pre-test to post-test) as revealed in one-samples *t*-tests.

**Fig 3:**
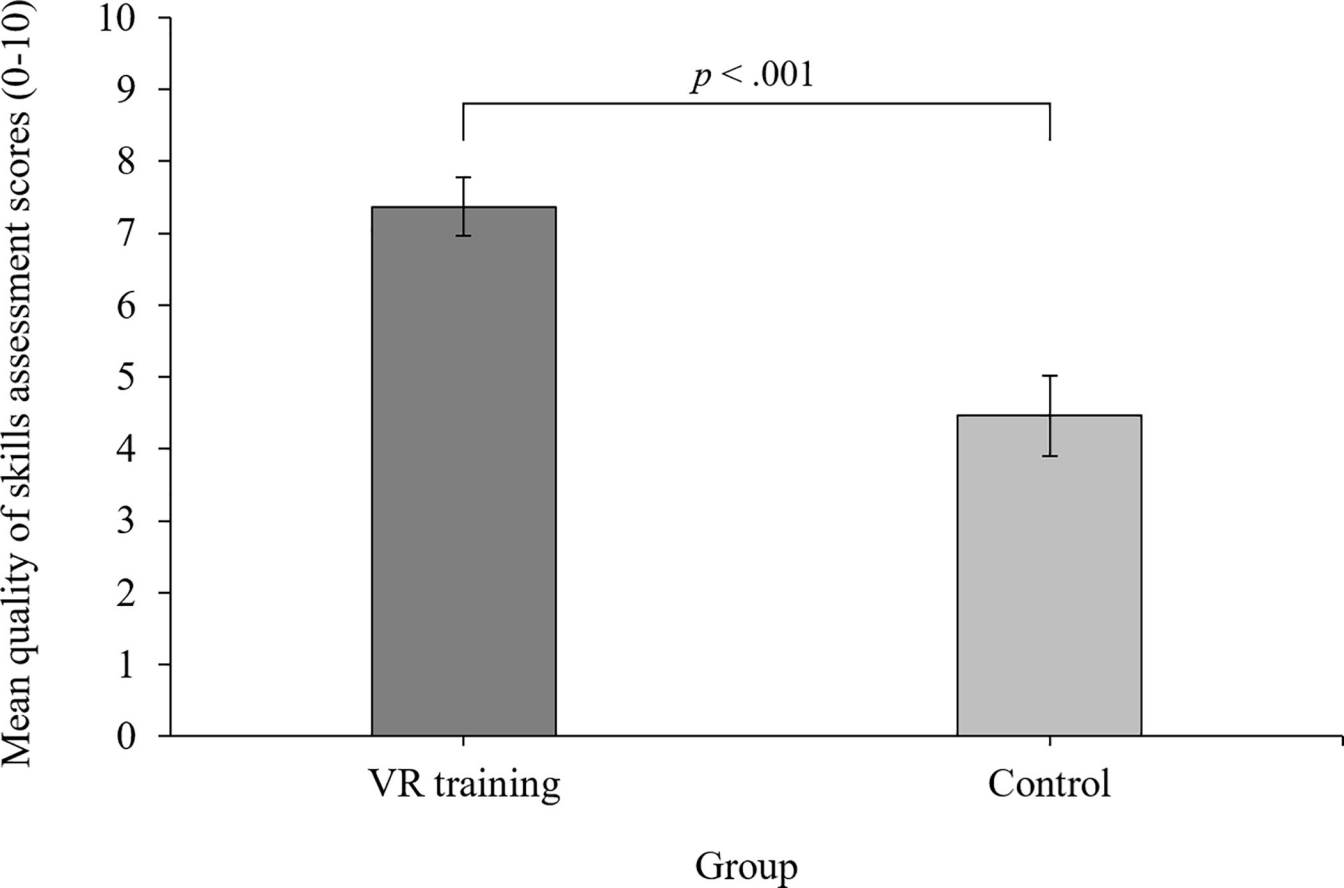
Quality of skills assessment. Total mean (± standard error) change scores on the quality of skills assessment for the VR training group (dark grey) and control group (light grey) from pre-test to post-test.

The mean change in participant’s scores from pre-test to post-test by group and the overall difference between groups on the quality of skills assessment, is displayed in Fig 3.

A Pearson correlation revealed that there was strong positive correlation in the participant’s change scores in the quantitative assessment and the quality of skills assessment, *r*(55) = .74, *p* < .001. The relationship accounted for 54.6% of the variance.

## Discussion

The present study examined the transfer effects from VR table tennis training to real world table tennis performance. Consistent with the hypothesis, it was found that real-world table tennis performance significantly improved on both quantitative aspects and overall skill quality, as compared to the no-training control group. These findings add to a sparse yet expanding literature regarding the use of VR as a tool to acquire and develop sports skills.

As this is one of the first studies to investigate transfer of training from VR in sports, it was necessary to first establish that the benefit was at least greater than no training. The current findings suggest that there is value in using VR as a complementary tool for training, especially for situations in which training is logistically difficult to organise or impractical in the real world. Future research can broaden the scope of the present findings by considering VR for other skill levels and training environments.

An interesting observation is that participants in both groups improved from pre-test to post-test. While 93.1% (27/29) of participants in the VR training group improved on the quantitative assessment, 85.7% (24/28) of participants in the no-training control also improved. The control group were not exposed to any form of table tennis training, as measured by self-report. A possible explanation as to why the control group significantly improved could be due to the repeated exposure of the assessment, being more familiar with the instructions and having time to think about how to best approach the task. This highlights the importance of having a control group as it is unclear why the participants improved while not undertaking any formal training.

Learner characteristics are known to affect training outcomes [23]. One important characteristic to consider concerning sports training is participant’s prior level of experience in the sport being assessed. However, all participants in the present sample were novice table tennis players. Thus, this study cannot answer whether VR training would also be effective in advanced and competitive players. Although the physics of the game may seem realistic to novices, it cannot be ruled out that competitive players would pick up slight differences in the timing of strokes, bounce, spin, and connection of a ball between the VR and real-world game. If this were the case, it is imaginable that training could even result in negative efficacy or poor transfer to the real world for competitive players [6, 23]. Moreover, it would also be beneficial to study other individual characteristics that are known to influence transfer such as cognitive ability, motivation, and personality [24].

In the present study, participants were not competitive table tennis players, nor did they receive any tips or insight on how to improve their skills. In future, it could be advantageous for participants to receive individualised coaching from the assessor on how to improve. In considering the application of VR into real-world practice, some researchers have suggested VR may be most valuable when used as a complementary training device for those who have inherent knowledge and experience in the sport [25]. Potentially, this is when VR will have the greatest benefits as a tool for training, when users can apply the correct form and technique in a realistic gameplay setting. Future research may gain insight into who would benefit the most from training in virtual environments by investigating people of varying skill levels.

The training tool used in the present study may have been so successful because of the effective combination of adaptive and open skill training in VR. Separately, adaptive training [8] and open skill training [14] have been shown to benefit performance outcomes, therefore we propose that a combination of adaptive and open skills training had a great impact on shaping participants skill development in VR. Taking into account that table tennis is primarily an open skills sport, we thought it was essential participants had the opportunity to train such skills in VR. Given the VR training comprised competitive games against AI, it would have been beneficial to determine if participants also became better players in real-world competition. However, this was beyond the scope of the present study.

Our study is one of the first to investigate the transfer of sports training from VR using a HMD. We were able to demonstrate transfer in a VR training group compared to a no-training control group; however, several factors could be considered for further investigation. The quantitative and quality of skill assessments tapped into a range of table tennis skills to measure change in participant’s abilities, but they did not assess all table tennis shots (e.g. drive, flick, smash) and were not based on objective measures such as video-based movement trajectory [17] or eye movement measurements [26]. In addition, we could not compare the effectiveness of VR sports training compared to real-world training because our study did not include a real-world training group. While these points could be addressed to build a more comprehensive assessment in future research, the measures employed in this study allow for concluding that training in VR can be used to improve fundamental table tennis skills in novices.

As new tests were developed to assess transfer in this study, the validity and reliability of the measures should be considered. The table tennis tests were constructed in consultation with experts from Table Tennis South Australia to ensure content validity. The tests also have high face validity as they measure what they purport to measure (i.e. real-world table tennis skills). The quantitative measures of the tests show good test-retest reliability as indicated by the intraclass correlation coefficient of 0.89. A limitation of our study design is that we cannot comment on the interrater reliability of the qualitative assessments. We decided to use one highly experienced table tennis coach to ensure consistency in the qualitative scoring of table tennis skills. We therefore do not know to what degree other assessors would agree with our assessor’s judgement of skills quality. Thus, while the scores of the quantitative assessment show good reliability, the generalizability of the qualitative findings may be limited.

Although we found positive transfer effects in the VR training group, it is uncertain what is driving these effects. As the results of the VR training group were compared to a no-training control, it is unknown if the improvement found was a direct outcome from the intended training, or whether the improvements were related to development in broader cognitive functioning, such as faster reaction time and better hand-eye coordination. Future research may benefit by introducing a control group that receives VR training, but in a similar table sport. For example, comparing VR table tennis training to a control group that trains with another table sport, such as VR air hockey. The purpose being, to keep variables consistent except the movement related to the activity. Comparing VR table tennis to another VR table sport could identify whether improvements are driven by the table tennis simulation or unspecific skills trained in VR—which are important in table tennis such as hand-eye coordination. Identifying the factors associated with improvements in real-world table tennis may provide insight into whether there is added value in developing sports applications for specific training in VR.

## Conclusion

This study adds to a sparse yet expanding literature demonstrating real-world transfer from VR sports training. In the present study, we found that real-world table tennis performance significantly improved on all performance measures after VR training as compared to no training. The present study is one of the first to investigate whether open skills can transfer to the real world in a sporting context, encouraging future research. The next step should be to investigate whether improvements in VR correlate with improvements in the real world, if VR is equal to, or better than real-world training, if VR training is beneficial for people of all skill levels and what is driving the effects behind VR training. It should then be considered whether transfer is limited to table tennis or whether training in a fast-paced sport also leads to real-world improvements in other sports. In an area that is largely unexplored, it appears VR is not limited to a tool that only fulfils entertainment purposes, but also has the potential to aid in the development of real-world skill and performance.
